# Pioneers and Pathfinders: 10 years of Frontiers in Medicine from t(ether)ed in-person medicine to untethered telemedicine

**DOI:** 10.3389/fmed.2026.1600039

**Published:** 2026-04-02

**Authors:** Ata Murat Kaynar

**Affiliations:** 1 Department of Anesthesiology and Perioperative Medicine, University of Pittsburgh, Pittsburgh, PA, United States; 2 The Center for Innovation in Pain Care (CIPC), University of Pittsburgh, Pittsburgh, PA, United States; 3 Department of Critical Care Medicine, University of Pittsburgh, Pittsburgh, PA, United States; 4 The Clinical Research, Investigation, and Systems Modeling of Acute Illness (CRISMA) Center, University of Pittsburgh, Pittsburgh, PA, United States

**Keywords:** telemedicine, pulse oximeter, COVID-19, central sensitization, troponin

## The last 10 years

Dr. Laennec is renowned for inventing the stethoscope, which tethered the physician to the patient (1, 2). A couple of decades later, Dr. Morton was recognized for demonstrating the effects of ether as a surgical anesthetic (3). Since the initial publication on the use of ether, the field of anesthesiology has had 180 years to evolve, experiencing an enormous expansion of the profession beyond the four walls of the operating room. The pre-cordial stethoscope of the anesthesiologist became the tether between the patient and the doctor over the years to ensure that the patient was breathing and had a healthy heartbeat under general anesthesia. Over time, this practice evolved into modern monitoring technologies such as pulse oximetry and capnography (4–7).

Similar to other medical fields, advancements in technology have made patient monitoring more accessible and standardized while also enabling remote monitoring and reducing the need for direct tethering—the new “untethered” care. The COVID-19 pandemic further accelerated this shift, popularizing telemedicine and other remote care models (8). Although these innovations have improved patient safety and accessibility, they have also distanced physicians from direct patient interaction, reshaping the nature of medical care, the patient-physician relationship, and trust.

In this decennial issue of *Pioneers and Pathfinders: 10 years of Frontiers in Medicine*, I would like to highlight manuscripts in anesthesiology and intensive care medicine that broaden our understanding and push the boundaries of patient care while still maintaining that close-knit, tethered, sacrosanct relation between the patient and the doctor.

Untethered care and telemedicine in anesthesiology: the preoperative patient assessment has been studied at the University of Buffalo, with high levels of confidence in the appropriate risk assessment of patients (9). These data have been supported by a multicenter survey among members of the European and American anesthesiology societies, who reported feeling comfortable using such technologies for perioperative risk assessment (8).

Racial bias in pulse oximetry measurement (10): University of Michigan researchers studied two large cohorts of critically ill patients requiring supplemental oxygen therapy. In their analysis, Black patients had nearly three times the frequency of occult hypoxemia that was not detected by pulse oximetry compared to white patients. These inherent biases in monitoring highlight the need to study various clinically important variables before widespread use, as important clinical decisions are made based on these data points.

Central sensitization: implications for the diagnosis and treatment of pain (11). This comprehensive review, published in *Pain*, has been highly cited for its in-depth analysis of central sensitization theories and its relevance to pain management, upon which new therapies are built.

Association between postoperative troponin levels and 30-day mortality among patients undergoing non-cardiac surgery (12). Although anesthesiologists are frequently asked to evaluate and mitigate perioperative cardiac event risks, myocardial injury marker-based assessments usually occur after an index event, such as an ischemic symptom. Two studies using troponin and high-sensitivity troponin serial measurements suggested that asymptomatic events do occur and correlate—as expected—with the risk of mortality. Featured in *JAMA*, the first study highlighted the significance of monitoring postoperative troponin levels to predict 30-day mortality in non-cardiac surgical patients. Patients with peak TnT values of 0.01 ng/mL or less, 0.02 ng/mL, 0.03–0.29 ng/mL, and 0.30 ng/mL or greater had 30-day mortality rates of 1.0%, 4.0%, 9.3%, and 16.9%, respectively. Peak TnT measurement added incremental prognostic value in discriminating those likely to die within 30 days when comparing models with and without peak TnT measurement. Similar findings were reported in a 2017 study that used high-sensitivity troponin measurements in a similar cohort of patients (13). Although these tests do suggest a risk of mortality, the clinical question remains whether to implement such expensive tests systemwide, which requires further studies.

Electroencephalogram signatures of loss and recovery of consciousness from propofol (14). The human experience of *consciousness* has its philosophical roots embedded in history, and Darwin proposed theories relating it to the evolutionary tree (15). The fear of being awake under general anesthesia is not a novel concept and has reached the mainstream media (16, 17). Published in the *Proceedings of the National Academy of Sciences*, research from MGH provided insights into the neural mechanisms of anesthesia-induced unconsciousness using the commonly used anesthetic agent propofol (14). This descriptive and mechanistic study does pave the way for us to have a better understanding of the transitions from consciousness to unconsciousness under general anesthesia. In theory, these EEG signatures could be used to assess other agents or levels of consciousness.

Old and frail requiring surgery (18). In a world where the population is aging, sometimes not in a healthy way, perioperative risks for patients are further increased.

The aging brain: age-dependent changes in the electroencephalogram during propofol and sevoflurane general anesthesia (19). This study, appearing in the *British Journal of Anaesthesia*, examined how aging affects brain activity under general anesthesia, offering valuable information for tailoring anesthetic care to older patients.

The safety of addition of nitrous oxide to general anesthesia in at-risk patients having major non-cardiac surgery (ENIGMA-II): a randomized, single-blind trial (20). Dating back to the time of Queen Victoria of England, nitrous oxide (aka *nitrous*) has had an up-and-down popularity in the scientific community, especially among anesthesiologists (21). Published in *The Lancet*, this large-scale ENIGMA-II trial assessed the safety of using nitrous oxide in general anesthesia for at-risk patients, influencing anesthesia practices worldwide by showing the safety of nitrous oxide in an era when other anesthetic agents have a negative impact on the environment.

Spinal anesthesia or general anesthesia for hip surgery in older adults (22). In this multicenter prospective trial comparing spinal anesthesia and general anesthesia for older patients undergoing hip surgery (795 spinal vs. 805 general anesthesia), spinal anesthesia was not superior to general anesthesia in mortality or other morbidity endpoints, including postoperative mobility.

Anesthetic depth and delirium after major surgery: a randomized clinical trial (23). Another common question posed by practicing anesthesiologists is the risk of postoperative delirium and the factors contributing to it. In this multicenter trial, 655 at-risk patients undergoing major surgery across three countries were assessed for delirium for 5 days postoperatively using the 3-min diagnostic confusion assessment method (3D-CAM) or CAM-ICU, and cognitive screening was performed using the mini-mental state examination at baseline and discharge, along with the Abbreviated Mental Test Score (AMTS) at 30 days and 1 year. There was no difference in the rate of delirium between patients with a BIS score of 50 and those with a BIS score of 35. However, patients in the BIS 50 group (i.e., with *a lighter level of anesthesia*) experienced a reduced risk of postoperative delirium and cognitive impairment at 1 year.

As it relates to science beyond the walls of the operating room, the following research is also of interest.

A randomized trial of protocol-based care for early septic shock (ProCESS trial) (24). This study evaluated protocol-based resuscitation in patients with early septic shock and found no significant difference in mortality compared to usual care, challenging previous early goal-directed therapy protocols (25).

Hydrocortisone plus fludrocortisone for adults with septic shock (APROCCHSS trial) (26). This trial demonstrated that the combination of hydrocortisone and fludrocortisone reduced mortality in patients with septic shock, supporting the use of corticosteroid therapy in this population.

Balanced crystalloids vs. saline in critically ill adults (SMART trial) (27). The study found that using balanced crystalloids, instead of saline, reduced the incidence of major adverse kidney events in critically ill adults, influencing fluid resuscitation practices and building further on the earlier research by the ANZICS group that compared albumin to saline (28).

Effect of a buffered crystalloid solution vs. saline on acute kidney injury among patients in the intensive care unit (SPLIT trial) (29). This trial assessed the impact of various resuscitation fluids on the risk of acute kidney injury (AKI). Comparing buffered crystalloid solutions to saline in ICU patients, the study found no significant difference in the rate of AKI, contributing to the ongoing debate on optimal fluid choice.

Comparing outcomes in patients with exsanguinating injuries: an Eastern Association for the Surgery of Trauma (EAST) multicenter, international trial evaluating prioritization of circulation over intubation (CAB over ABC) (30). A common observation among intensivists and emergency physicians is that the intubation process, along with the associated induction agents and the removal of sympathetic drive upon relief of respiratory distress, can lead to hemodynamic instability. This question was studied by the research group in a multicenter trial, which suggested that prioritizing circulation before intubation is associated with improved outcomes in patients with exsanguinating injuries, potentially reshaping trauma resuscitation protocols.

Prehospital plasma during air medical transport in trauma patients at risk for hemorrhagic shock (31). Regarding the choice of resuscitation fluid, blood and blood products have fallen out of favor in perioperative medicine. However, this study, focusing on trauma patients at risk of hemorrhagic shock, showed that pre-hospital use of thawed plasma improved 30-day mortality compared to the standard of care, challenging the current paradigms.
